# Development and Validation of the Professional Fit Scale in Nursing

**DOI:** 10.1002/nop2.70309

**Published:** 2025-11-19

**Authors:** Tuba Çatak, Betül Sönmez

**Affiliations:** ^1^ Department of Nursing, Faculty of Health Sciences Istanbul Gedik University Istanbul Türkiye; ^2^ Department of Nursing Management, Florence Nightingale Faculty of Nursing Istanbul University‐Cerrahpaşa Istanbul Türkiye

**Keywords:** nurse, professional fit, reliability, scale development, validity

## Abstract

**Aim:**

This study aims to develop the Professional Fit Scale in Nursing and to test psychometric analyses.

**Design:**

This is a scale development and validation study.

**Methods:**

This study was conducted in three phases: (1) creation of the items, (2) preliminary assessment of the items, and (3) assessment of the psychometric properties of the scale. The scale was tested using construct validity and reliability analyses after the assessment of content and face validity in accordance with the scale development guidelines. The data were collected between May and September 2021 from nurses working in Istanbul (*n* = 720).

**Results:**

The final scale included 56 items and 9 subscales, explaining 68.06% of the total variance. CFA showed acceptable fit (*χ* ^2^/df = 2.57; GFI = 0.90; CFI = 0.90; RMSEA = 0.05; RMR = 0.07). Factor loadings ranged 0.592–0.922. Convergent (CR > 0.70; AVE > 0.50) and discriminant validity (MSV < AVE; ASV < MSV) were supported. Internal consistency was high (*α* = 0.97), and test–retest reliability was strong (ICC = 0.99).

**Conclusion:**

The scale, which was developed, evaluates the professional fit of nurses in nine fields: professional responsibility, interpersonal relations and care, problem solving, coping with stress, professional skills, continuous development, working conditions, professional achievement and professional autonomy.

**Implications for the Profession and Patient Care:**

Assessing the professional fit of nurses can contribute to the effective management of the healthcare workforce, increase the quality of nursing services and patient safety, which in turn may increase patient and community satisfaction.

**Impact:**

- The scale is the first tool to measure nurses' professional fit.
- The scale demonstrates strong reliability, validity and psychometric properties.
- The developed scale can be used in identifying job mismatches and implementing improvements, career planning and shaping human resources policies.

**Patient or Public Contribution:**

Patients or members of the public were not included in the study.

## 1 Introduction

Person‐occupation fit, a key concept in occupational psychology (Glosenberg et al. 2019), represents one of the various dimensions of person‐environment fit, alongside person‐job, person‐organisation, person‐team, and person‐supervisor fit. It describes the alignment between an individual's characteristics and values and the profession's expectations and requirements, such as the necessary skills and competencies (Vogel and Feldman 2009). Person‐occupation fit is considered a predictor of performance and job satisfaction by researchers and practitioners, particularly in many fields of organisational and occupational psychology (Hoff et al. 2020), and is often used to predict various workplace attitudes and behaviours (Kwantes et al. 2012). According to various research results, it has been found that occupational fit reduces the perceived stress of employees and increases their job satisfaction, organisational commitment, and person‐organisation fit, respectively (Vogel and Feldman 2009). Accordingly, when nurses experience a high level of fit between their personal characteristics (such as skills, job values and style) and their professional environment, they may experience greater job satisfaction and be more likely to stay in the profession. This fit can also improve the quality of care and patient outcomes (Kutney‐Lee et al. 2013). However, today, nurses are not satisfied with their profession due to the lack of adequate staff and resources, having to work under adverse working conditions such as heavy workload (Yu et al. 2021), and may leave their profession in the first years of their career (Blay and Smith 2020). This situation leads to the inability to eliminate the global issue of nurse shortage (Samoya et al. 2015). The World Health Organization [WHO] (2020) highlights the current situation of the global nursing workforce in the World Nursing Situation Report, which includes data from 191 countries, including Turkey, and has reported that the global nurse shortage is about 6 million. The current situation underscores the vital need for strategic investments in the global nursing workforce to improve health outcomes and to achieve broader public health goals. In this direction, determining the occupational fit of nurses, which is the subject of this study, may be a basic strategy for empowering nurses and taking proactive measures for the nursing workforce.

## 2 Background

Occupational fit has been considered a key concept in Person‐Environment Fit Theory (Van Vianen 2018), occupational choice theories (for instance, The Theory of Work Adjustment (Dawis and Lofquist 1984) and Holland's Occupational Typology (Holland 1997)), and each focuses on the need for individuals to be matched with suitable professions to ensure positive outcomes (Glosenberg et al. 2019). The concept of occupational fit in nursing is closely related to the development of professional identity. Professionalism in nursing refers to the value orientations and standards that reflect the nursing concepts and work attitudes of nurses who practice the nursing profession (Xue et al. 2023). According to a qualitative research study conducted on American nurses, establishing professional identity in nursing is based on the fit of one's values and skills with the values of their colleagues and the environment in which they work (Ranjbar et al. 2017). Consequently, occupational fit in nursing plays an important role in the implementation of professional nursing and the overall quality of patient care.

Researchers have used different methods to evaluate person‐occupation fit. Some researchers measure fit by focusing on the main interests of individuals, while other researchers have included more personality variables to compare the person with the occupation. In general, occupational fit studies lack sound methodologies that are necessary to test the effects of fit and have been used in other fields (Van Vianen 2018). The measurement of the fit that people perceive with their occupation is carried out using two methods: directly (by asking people how compatible they are with their occupation) or indirectly. However, it is stated that direct fit measurements provide a more holistic and consistent assessment compared to indirect measurements and are the best to measure the size of fit in occupational psychology (Kristof‐Brown et al. 2005). In this research, a direct evaluation approach has also been adopted for fit measurement.

Various tools have been developed to evaluate person‐job and person‐occupation fit (Brkich et al. 2002; Kusluvan and Kusluvan 2000; Nota et al. 2012; Saks and Ashforth 2002; Ültanır 2001; Yılmaz and Tanrıverdi 2017; Weng and McElroy 2010). These measurement tools have been developed on non‐nursing professions to determine the suitability of individuals for a profession or job with a small number of items. However, professions should be treated separately since they have a wide variety of differentiating characteristics (Saks 2016). Despite their importance, there is no universally recognised scale specifically designed to measure occupational fit in nursing. However, several scales have been developed that serve to evaluate some aspects related to occupational fit, including professional values, professional competencies and the perceived nursing work environment. These include the Nurses Professional Values Scale‐3 (NPVS‐3), which measures the professional values that are very important for occupational fit among nurses (Weis and Schank 2017); Nursing Competence Scale that provides insights into the professional competence aspects (Meretoja et al. 2004); Professional Nurse Self‐Assessment Scale (ProffNurse SAS) for the self‐assessment of clinical competence of nurses (Finnbakk et al. 2015); Nurse‐Work Instability Scale which estimates potential job instability by evaluating the fit between the nurse's abilities and job requirements (Gilworth et al. 2007); and the Nursing Work Index‐The Practice Work Environment Scale, which is used as an indicator of the working atmosphere in nursing environments and plays a fundamental role in measuring occupational fit (Lake 2002). These measurement tools do not directly measure the occupational fit of nurses and can only be used to understand certain aspects.

In this study, the Trait‐Factor Theory and the Content Model, which form the conceptual basis of Occupational Information Network (O*NET), were used to sort the occupation‐specific requirements (for instance, basic skills, skills, job values and work style) and predict the occupational fit levels of nurses. The Trait‐Factor Theory emphasises the importance of matching individuals' attitudes and behaviors, interests, values, personality, requirements, and the conditions and requirements of the profession to achieve positive outcomes (Glosenberg et al. 2019). O*NET is a public resource created as a result of a large‐scale research project supported by the United States (USA) Department of Labor and widely used internationally (Glosenberg et al. 2019). It provides information for more than 900 professions, stores this information in a comprehensive database (https://www.onetcenter.org/overview.html), which is collected from three main sources: officials, professional specialists and professional analysts (Burgoyne et al. 2021). The O*NET Content Model was developed using Dawis and Lofquist's Theory of Work Adjustment (Dawis and Lofquist 1984) and Holland's Occupational Typology (Holland 1997), and includes identifiers arranged in six areas (employee characteristics, employee needs, experience requirements, occupation‐specific information, workforce characteristics and job requirements) that determine the basic qualities and characteristics of employees and professions (https://www.onetcenter.org/content.html). Consequently, based on the propositions in the Trait‐Factor Theory and the nursing‐specific definitions in the Content Model, which form the conceptual basis of O*NET, we conceptualised the occupational requirements of nurses within the framework of 4 theoretical components: (1) basic abilities, (2) skills, (3) work values and (4) work style.

## 3 The Study

### 3.1 Aim and Design

The aim of this scale development and validation study is to develop the ‘Professional Fit Scale in Nursing’ to determine the professional fit levels of nurses and to test their psychometric properties.

### 3.2 Methodology

**FIGURE 1:**
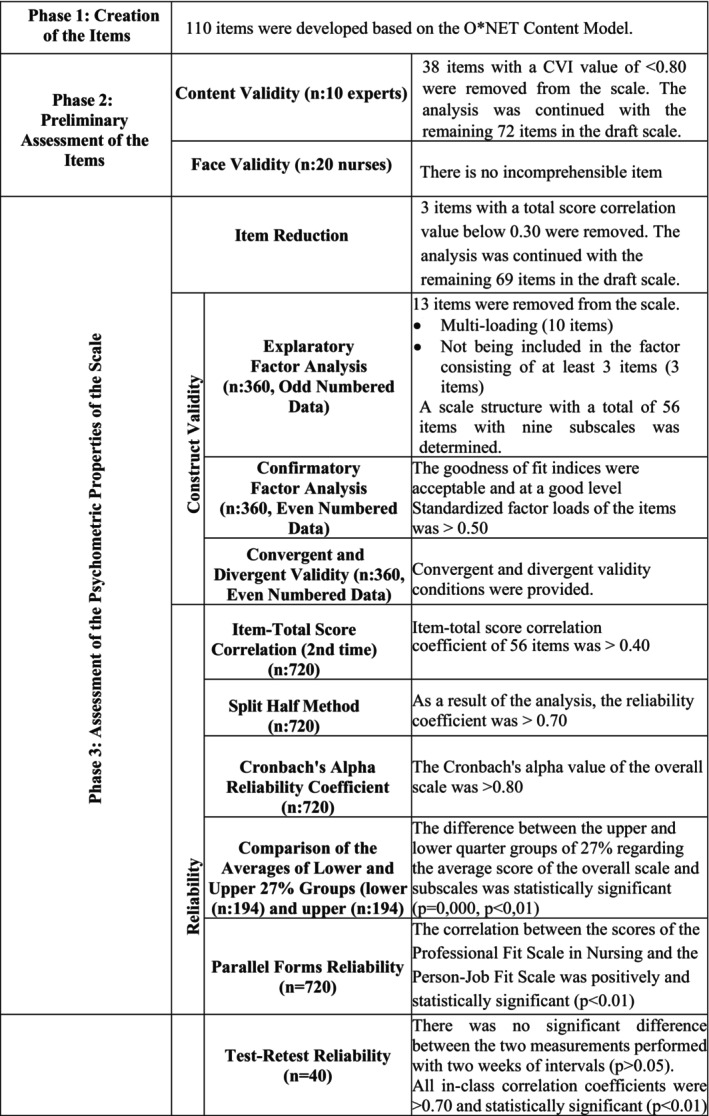
Phase of development of the Professional Fit Scale in Nursing.

This study followed internationally recognised guidelines for the development and validation of psychometric instruments, particularly those proposed by Boateng et al. (2018) and Carpenter (2018). In line with these frameworks, the scale development process was conducted in a three‐phase design: (1) creation of the items, (2) preliminary assessment of the items and (3) assessment of the psychometric properties of the scale, including validity and reliability analyses (Figure 1). Furthermore, the psychometric stages were reviewed and structured according to the Consensus‐based Standards for the Selection of Health Measurement Instruments (COSMIN) risk of bias checklist (Appendix S1) (Mokkink et al. 2024). The study was conducted between August 2020 and September 2021.

#### 3.2.1 Phase 1: Creation of the Items

In order to create the draft scale, the scales, which were developed to determine the identifiers arranged in six main areas for registered nurses (employee characteristics, employee needs, experience requirements, occupation‐specific information, workforce characteristics and job requirements) in the O*NET Content Model, and certain aspects of the professional fit of the nurses mentioned above (Finnbakk et al. 2015; Gilworth et al. 2007; Lake 2002; Meretoja et al. 2004; Weis and Schank 2017) were examined in detail.

Although in the specific context of the USA, in our study, we based our findings on the occupation‐specific identifiers specified for registered nurse in the O*NET Content Model. Because nurses with the title of registered nurse are nurses who have a bachelor's degree, as in many countries. In addition, as far as is known, O*NET is the only reliable and up‐to‐date international source of international information about professional characteristics and employee qualifications in a wide range of professions (Glosenberg et al. 2019), and different researchers have used the O*NET database in their studies to develop scales or questionnaires (for instance, Morgeson and Humphrey 2006; Phan and Rounds 2018).

The initial item pool was developed by the authors who had clinical experience and an academic background. The first author was primarily responsible for drafting the items based on the O*NET Content Model, theoretical framework and literature review. The writing process was conducted collaboratively to ensure both content accuracy and conceptual clarity. Because this study was part of a doctoral dissertation, the item development process was reviewed by the thesis monitoring committee consisting of two nursing faculty members other than the advisor. These individuals reviewed the draft items and provided feedback on clarity, appropriateness and consistency with the theoretical framework.

Accordingly, the employee (person) requirements for the requirements of nursing (occupation) were determined, and a 110‐item pool was created under four main dimensions (basic abilities, skills, work values and work style). The items were written in present tense, and 3 of them had negative meanings. It was decided to respond to the draft scale items in a 5‐point Likert type (1: Strongly disagree, 2: Disagree, 3: Neutral, 4: Agree and 5: Strongly agree) to provide a wide range of options and increase variability (Polit and Beck 2017).

#### 3.2.2 Phase 2: Preliminary Assessment of the Items

##### 3.2.2.1 Content Validity

The items developed in the first stage were submitted to the opinion of 10 experts for content validity. The experts consisted of academicians with doctorates in the fields of nursing fundamentals (4 experts), psychiatric nursing (2 experts), nursing management (3 experts), and human resources management (1 expert). They evaluated the content validity of each item based on a 4‐point Likert scale as ‘1 = Not suitable at all, 2 = Major revision is required, 3 = Minor revision is required and 4 = Completely suitable’. They were also asked to submit their suggestions, if any, to ensure comprehensibility. As a result of the expert opinions, 38 items with a CVI of < 0.80 were removed from the draft scale. The CVI of the remaining 72 items was found to range between 0.80 and 1.00, while the CVI of the overall scale was found to be 0.84.

##### 3.2.2.2 Face Validity

After content validity, the draft scale was applied online to 20 nurses who were not included in the main study sample but shared similar characteristics (e.g., education level, clinical experience) with the target group in order to assess face validity. These nurses were selected through convenience sampling and were known to the researchers. Data collection was carried out via an online self‐administered questionnaire. After completing the draft scale, participants were asked to provide feedback on any items they found unclear, confusing or difficult to interpret. The feedback was based on the subjective perceptions of the participants, so no structured rating scale was used; the evaluation was qualitative in nature. All participants reported that the items were clear and comprehensible. Since no issues were raised, no further revisions were made at this stage, and the scale proceeded to the next validation phase (Boateng et al. 2018).

#### 3.2.3 Phase 3: Assessment of the Psychometric Properties of the Scale

##### 3.2.3.1 Item Reduction

At this stage, the item‐total score correlation of the draft scale and Cronbach's alpha values when the item was deleted were examined. As a result of the analysis, items with a low correlation value (< 0.30), whose removal from the scale would increase the overall Cronbach's alpha coefficient, were removed from the draft scale, respectively (Boateng et al. 2018). The item‐total score correlation values of the remaining 69 items were found to range between *r* = 0.32 and *r* = 0.80.

##### 3.2.3.2 Construct Validity

Construct validity of the draft scale was assessed in different sample groups using the exploratory factor analysis—EFA (*n* = 360, data with odd number), confirmatory factor analysis—CFA (*n* = 360, even‐numbered data) and convergent and divergent validity analysis (*n* = 720, all data). Validity analyses started with EFA. Prior to the EFA, Kaiser‐Meyer‐Olkin (KMO) and Bartlett's sphericity tests were applied to the data set to assess the applicability of the analysis. Principal Component Analysis and varimax rotation method, which are frequently used due to easy‐to‐practice interpretation, were used to determine the factor structure of the draft scale, and the eigenvalue method and scree plot were used to determine the number of factors (Büyüköztürk 2020). CFA was performed to test the validity of the scale structure revealed in the EFA. In CFA, different fit indices, standardised factor loadings, and the results of convergent and divergent validity analyses were examined (Büyüköztürk 2020). The average variance (AVE; average variance extracted, the preferred value > 0.50) and reliability (CR; Composite reliability, the preferred value > 0.70) were calculated for the convergent validity based on the factor load values. In addition to AVE and CR values, maximum squared variance (MSV) and the average shared variance (ASV) values were calculated for divergent validity (preferred value MSV < AVE; ASV < MSV) (Yaşlıoğlu 2017).

##### 3.2.3.3 Reliability

In this stage, the item analysis of the scale was redone after the construct validity. In addition, the split‐half method, Cronbach's alpha internal consistency coefficient, *t*‐test in independent groups (upper and lower groups of 27%), parallel form reliability and test–retest analysis results were examined. In this study, a Cronbach's alpha value above 0.70 for the subscales, and the overall scale was considered an indicator of good internal consistency (Büyüköztürk 2020). The Person‐Job Fit Scale, developed by Brkich et al. (2002) and adapted to Turkish by Kerse (2018), was used to determine the parallel form reliability of the draft scale. In order to assess over‐time stability, the draft scale was applied to a sample of 40 nurses with an interval of 15 days, and the correlation between test–retest results was assessed using the dependent groups *t*‐test and intra‐class correlation coefficient (ICC).

### 3.3 Participants and Setting

The population of the research consisted of nurses working in health institutions in Istanbul. The inclusion criteria were as follows: (1) to be a nurse working in Istanbul, (2) to work actively during the data collection process and (3) to volunteer to participate in the research. In the scale development studies, it is stated that 5 or 10 times the number of scale items should be reached to evaluate the validity and reliability, and that the quality of the analysis improves as this ratio increases (Büyüköztürk 2020). Therefore, it was planned to reach 720 people (10 times the number of items, 72 × 10 = 720) according to the draft scale consisting of 72 items. A sample size of 300 is considered a good sample size for factor analysis (Alpar 2020), and it is recommended that EFA and CFA be performed in separate samples (Boateng et al. 2018). Therefore, EFA was conducted with 360 nurses (odd numbered data), while CFA was conducted with 360 (even‐numbered data), the test–retest stage was conducted with 40, and item reduction and internal consistency and correlation analysis stages were conducted with 720 nurses (all data).

### 3.4 Data Collection Tools

The Professional Fit Scale in Nursing‐Draft was used in data collection. The draft scale was based on self‐reporting and consisted of four main subscales and 72 items: basic ability (5 items), skill (22 items), work values (15 items) and work styles (30 items). Items were scored on a 5‐point Likert‐type scale (1: Strongly disagree, 5: Strongly agree). There were 3 items with negative expressions, which were reverse‐scored. The average completion time for the Professional Fit Scale in Nursing was approximately 10–12 min.

In addition to the draft scale, the Nurse Information Form, consisting of a total of 7 questions related to research variables, questioning the socio‐demographic and professional characteristics of nurses, was used.

In the third stage of the research, the Person‐Job Fit Scale, developed by Brkich et al. (2002), adapted to Turkish by Kerse (2018), was used to determine the parallel form reliability of the draft scale. The scale provides an assessment of the extent to which an individual's knowledge, skills, abilities, needs and values coincide with professional requirements based on self‐reporting. It consists of nine items and a single dimension rated on a 5‐point Likert‐type scale (1: Strongly disagree, 5: Strongly agree), and the assessment is performed based on the score average. Four of the scale items are reverse‐scored. The Cronbach's alpha of the original scale (Brkich et al. 2002) and the Turkish version is 0.91 (Kerse 2018).

### 3.5 Data Collection

The data were collected online between May and September 2021 due to the COVID–19 pandemic. In this process, a Google Form containing the data collection tools was created by the researchers, including the data collection tools (Professional Fit Scale in Nursing‐Draft, Person‐Job Fit Scale and Nurse Information Form). The link to the form was sent to nurses via e‐mail and social media applications using convenience and snowball sampling methods.

### 3.6 Data Analysis

For data analysis, IBM SPSS 22.0 and AMOS 21 software were utilised. Descriptive statistical methods, including frequency, percentage, mean and standard deviation, were employed to examine the characteristics of the participants and their scale scores. Additionally, Microsoft Excel was used to process the data collected from expert evaluations. The validity and reliability of the scale were assessed through the analytical methods outlined in the methodology section of this study.

### 3.7 Ethical Considerations

Permission was obtained via email from the author (G. Kerse) who adapted the Person‐Job Fit Scale to Turkish, which was used to assess the parallel form reliability in this study. Ethics committee approval was obtained from the Istanbul University‐Cerrahpaşa Social Sciences and Humanities Research Ethics Committee (Date: 07.09.2020, Issue: 53728, Decision No: 2020/125). All procedures in the study performed comply with the 1964 Declaration of Helsinki and its later amendments. An Informed Consent Form was included in the Google Form in addition to the information on the purpose of the research, its duration and the actions to be performed throughout the study. The statement ‘I am working in Istanbul’ was included as a mandatory response option to ensure that the questionnaire would be filled out only by those working in Istanbul. The research data were collected from the nurses who agreed to participate in the study and approved the online Informed Consent Form.

## 4 Results

### 4.1 General Characteristics of the Participants

The average age of the 720 nurses participating in the study was 28.77 (SD = 6.90) (min 18, max 56), while the majority (82.4%, *n* = 593) were female, single (64.6%, *n* = 465) and bachelor's degree graduates (68.1%, *n* = 490). The average professional experience was 6.54 (SD = 7.05) (min 1, max 33), while those with 1–5 years of experience (63.6%, *n* = 458) constituted more than half of the participants. It was determined that 24.3% (*n* = 175) of the nurses were working in internal units, 17.8% (*n* = 128) in surgical units and 16.9% (*n* = 122) in the emergency department and the majority (63.9%, *n* = 460) were working in shifts (Table 1).

**TABLE 1:** Distribution of socio‐demographic and professional characteristics (*N*: 720).

Age (years)	Number (%)
Min–max (median)	18–56
Mean ± SD	28.77 ± 6.90
Gender	
Female	593 (82.4)
Male	127 (17.6)
Marital status	
Married	255 (35.4)
Single	465 (64.6)
Educational level	
Health vocational high school	83 (11.5)
Associate degree	52 (7.2)
Bachelor's degree	490 (68.1)
*Post‐graduate	95 (13.2)
Duration of professional experience (years)	
Min–max (median)	1–33
Mean ± SD	6.54 ± 7.05
Current unit	
Polyclinic	72 (10.0)
Internal medicine	175 (24.3)
Surgical	128 (17.8)
Emergency department	122 (16.9)
Intensive care	92 (12.8)
COVID‐19 unit	21 (2.9)
Operating room	41 (5.7)
Delivery room	16 (2.2)
Education and R&D	29 (4.0)
Family Health Center	24 (3.3)
Working style	
Constantly day shift	242 (33.6)
Constantly night shift	18 (2.5)
Shift work	460 (63.9)

*Note:* *indicates Post‐graduate refers to nurses with a master’s degree.

### 4.2 Construct Validity

After the content and face validity of the draft scale, the number of items decreased from 110 to 72. After the item analysis, 3 items were removed, and the 69‐item scale entered the factor analysis step.

In the factor analysis, the KMO value of the draft scale was 0.95 (> 0.60) and the Bartlett test (*x* ^2^ = 15,874.215; *p* = 0.000; *p* < 0.05) was found to be significant. It was determined that the sample size was adequate and suitable for factor analysis. As a result of the factor analysis performed on 69 items, a total of 13 items were removed from the scale, 10 items with a cross‐load of less than 0.10 in more than one factor, and 3 items that did not meet the criteria of having at least 3 items per factor (Boateng et al. 2018). The scale was finalised with nine subscales (factors) and a total of 56 items. EFA results showed that all nine factors had eigenvalues above 1, meeting Kaiser's criterion. In conclusion, the subscales of the Professional Fit Scale in Nursing‐Draft were determined to be named as ‘professional responsibility, interpersonal relations and care, problem solving, coping with stress, professional skills, continuous development, working conditions, professional achievement and professional autonomy’, respectively. These nine subscales explained 68.06% of the total variance. The high ratios showed that the scale had a strong factor structure (Table 2).

**TABLE 2:** Exploratory factor analysis of the Professional Fit Scale in Nursing (*n* = 360).

Item no.	Items	Factor loads
*Factor 1: Professional responsibility (eigenvalue = 24.126; explained variance = 15.457)*		
50	I make an effort to do my job right.	0.751
58	I consult others on issues that I am incapable of.	0.723
60	When working as a team member, I fulfill my responsibilities.	0.712
49	I'm careful when I do my job.	0.711
33	I am known as a reliable person.	0.680
48	I finish my tasks on time.	0.630
59	I can work together with members of different professions.	0.627
47	I can set priorities when I need to do multiple tasks at the same time.	0.616
34	The people I work with trust me.	0.597
38	I adhere to professional ethical principles.	0.594
36	I take responsibility for my professional practices.	0.567
54	I make an effort to be successful in my profession.	0.541
31	I think I am successful at my job.	0.521
37	My personal values are compatible with my professional values.	0.482
*Factor 2: Interpersonal relations and care (eigenvalue = 3.283; explained variance = 10.219)*		
11	I look for ways to help people.	0.734
10	I like helping people.	0.723
6	I can easily establish relationships with people.	0.671
7	My verbal communication skills are good.	0.659
9	I am aware of the needs of the people/people I provide care for.	0.654
12	I can evaluate the individual holistically.	0.633
8	My written communication skills are good.	0.602
13	I can provide nursing care in accordance with the individual's requirements.	0.556
*Factor 3: Problem solving (eigenvalue = 2.257; explained variance = 9.055)*		
19	I can develop different and creative solutions to problems.	0.694
17	I identify problems easily.	0.658
20	I adopt a proactive (preventive) approach to problem solving.	0.648
18	I solve problems rationally.	0.628
22	The decisions I make are mostly effective.	0.600
23	I can easily make difficult decisions in risky conditions.	0.575
27	I can convince others to change their mind or behaviour.	0.573
16	I easily notice possible risks in any environment/situation.	0.527
*Factor 4: Coping with stress (eigenvalue = 1.947; explained variance = 7.276)*		
65	I can manage my anger.	0.767
64	I effectively cope with stressful situations.	0.743
62	I can remain calm when I'm under pressure.	0.710
63	I am always open to negative feedback.	0.607
66	Continuous improvement is a philosophy of life to me.	0.535
67	I can adapt to constantly changing conditions.	0.522
68	I can take an active role in the change process.	0.504
*Factor 5: Professional skills (eigenvalue = 1.557; explained variance = 6.643)*		
3	I can use electronic devices or equipment related to my profession/job without difficulty.	0.738
4	I can easily use information and communication systems (hardware and software) related to my profession/job.	0.711
2	I can synthesise the information and integrate it into my professional practices.	0.682
1	I can easily learn new information.	0.624
5	I have adequate psychomotor skills to carry out my professional practices.	0.537
*Factor 6: Continuous development (eigenvalue = 1.488; explained variance = 6.300)*		
71	I closely follow the developments related to my profession.	0.821
70	I conduct research on my professional practices.	0.800
72	I try new ways/methods related to my professional practices.	0.705
69	I try to improve my professional competence.	0.553
*Factor 7: Working conditions (eigenvalue = 1.305; explained variance = 5.043)*		
52	I do not avoid working in a risky work environment.	0.804
51	I can work on a shift system.	0.711
46	It does not bother me to be constantly busy at my job.	0.683
53	I can work wearing personal protective equipment.	0.677
*Factor 8: Professional achievement (eigenvalue = 1.145; explained variance = 4.280)*		
28	I think that my profession meets my personal goals.	0.796
32	Nursing meets my career expectations.	0.712
29	My profession allows me to use the skills I have.	0.708
*Factor 9: Professional autonomy (eigenvalue = 1.007; explained variance = 3.789)*		
41	I take decisions on my authorised practices independently.	0.727
40	I would like to make decisions about my job myself.	0.657
42	I do my job without anyone else's supervision.	0.589
Total variance of the scale = 68.062%		

As a result of the CFA, the fit indices were found to be good fit or were at an acceptable level: Adjusted chi‐square statistics (*χ* ^2^/SD) = 2.57, The goodness of fit index (GFI) = 0.90, comparative fit index (CFI) = 0.90, mean square error of approximation (RMSEA) = 0.05, and root mean square residual (RMR) = 0.07. The standardised regression coefficients of all items were above 0.50 (between 0.592 and 0.922), and the convergent and divergent validity results were acceptable (CR > 0.70; AVE > 0.50; MSV < AVE; ASV < MSV). These results show that the items in the scale explain the factors at a high rate and consistently with each other, and that the dimensions in the model are separate constructs and discriminant validity is achieved (Table 3).

**TABLE 3:** The results obtained from the Confirmatory Factor Analysis and reliability analyses of the Professional Fit Scale in Nursing.

Items	Unstandardised factor loading *Β*	Standard error SE	*p*	Factor loading Std. β	CR	AVE	MSV	ASV	Cronbach's alpha
*Factor 1: Professional responsibility*					0.833	0.630	0.307	0.241	0.961
m37	1.000			0.734					
m31	1.038	0.066	< 0.001	0.797					
m54	1.001	0.063	< 0.001	0.805					
m36	1.120	0.064	< 0.001	0.877					
m38	1.046	0.062	< 0.001	0.853					
m34	0.986	0.063	< 0.001	0.792					
m47	1.035	0.065	< 0.001	0.814					
m59	1.072	0.064	< 0.001	0.850					
m48	0.947	0.061	< 0.001	0.791					
m33	1.112	0.064	< 0.001	0.880					
m49	1.060	0.062	< 0.001	0.864					
m60	1.127	0.068	< 0.001	0.840					
m58	1.069	0.063	< 0.001	0.852					
m50	1.062	0.063	< 0.001	0.848					
*Factor 2: Interpersonal relations and care*					0.969	0.688	0.641	0.634	0.949
m13	1.000			0.892					
m8	0.965	0.046	< 0.001	0.812					
m12	1.002	0.039	< 0.001	0.892					
m9	1.039	0.041	< 0.001	0.884					
m7	1.002	0.044	< 0.001	0.841					
m6	1.002	0.045	< 0.001	0.834					
m10	0.996	0.042	< 0.001	0.855					
m11	1.010	0.047	< 0.001	0.817					
*Factor 3: Problem solving*					0.956	0.729	0.702	0.590	0.924
m16	1.000			0.820					
m27	0.767	0.063	< 0.001	0.592					
m23	0.885	0.059	< 0.001	0.702					
m22	0.960	0.062	< 0.001	0.717					
m18	1.156	0.052	< 0.001	0.915					
m20	1.094	0.060	< 0.001	0.808					
m17	1.137	0.053	< 0.001	0.896					
m19	1.153	0.053	< 0.001	0.897					
*Factor 4: Coping with stress*					0.933	0.641	0.611	0.589	0.900
m65	1.000			0.744					
m64	0.981	0.065	< 0.001	0.772					
m62	0.959	0.080	< 0.001	0.630					
m63	0.963	0.068	< 0.001	0.734					
m66	1.137	0.069	< 0.001	0.839					
m67	1.124	0.065	< 0.001	0.878					
m68	1.076	0.064	< 0.001	0.851					
*Factor 5: Professional skills*					0.916	0.612	0.546	0.526	0.925
m3	1000			0.839					
m4	0.950	0.035	< 0.001	0.817					
m2	1016	0.043	< 0.001	0.922					
m1	1.067	0.050	< 0.001	0.872					
m5	0.909	0.044	< 0.001	0.856					
*Factor 6: Continuous development*					0.916	0.612	0.546	0.526	0.909
m71	1.000			0.897					
m70	1.048	0.040	< 0.001	0.901					
m72	0.929	0.043	< 0.001	0.826					
m69	0.957	0.039	< 0.001	0.880					
*Factor 7: Working conditions*					0.930	0.768	0.753	0.492	0.746
m52	1.000			0.711					
m51	0.916	0.092	< 0.001	0.591					
m46	0.862	0.083	< 0.001	0.616					
m53	0.967	0.078	< 0.001	0.765					
*Factor 8: Professional achievement*					0.768	0.548	0.527	0.415	0.802
m28	1.000			0.860					
m32	0.801	0.066	< 0.001	0.614					
m29	1.025	0.059	< 0.001	0.879					
*Factor 9: Professional autonomy*					0.833	0.630	0.307	0.241	0.839
m41	1.000			0.769					
m40	1.064	0.064	< 0.001	0.864					
m42	1.025	0.066	< 0.001	0.801					

As a result of correlation analysis, the overall Professional Fit Scale in Nursing score was found to have the strongest correlation with professional responsibility (*r* = 0.937; *p* = 0.001), and the weakest correlation with professional achievement (*r* = 0.548; *p* = 0.001). When the relationships between the subscales were examined, it was found that the strongest correlation was between professional responsibility and interpersonal relations and care subscales (*r* = 0.822), while the weakest correlation was between professional autonomy and professional achievement (*r* = 0.292). As a result, these findings obtained from the correlation analysis reveal that the overall and sub‐dimensions of the scale are consistent with each other and provide a valid measurement tool both theoretically and practically.

### 4.3 Reliability

After the construct validity, the item analysis of the scale was performed again. As a result of the analysis, it was seen that there was no item with a correlation coefficient below 0.30 (between 0.344 and 0.771). Cronbach's alpha value was 0.97 for the overall scale and 0.74–0.96 for subscales (Table 3).

Split‐half analysis showed that the reliability coefficients of both halves were above the acceptable level (> 0.70), and the correlation between the two halves (*r* = 0.804, *p* < 0.001) was significantly high (DeVellis and Thorpe 2021). According to the *t*‐test analysis in independent groups (27% upper and lower groups), the scale items provide measurements that distinguish individuals (*p* = 0.000, *p* < 0.01) (Büyüköztürk 2020). In the parallel form reliability analysis, it was confirmed that the scale provides parallel measures when used in conjunction with a scale measuring person‐job fit in general (*r* = 0.347, *p* < 0.05) (Boateng et al. 2018). In the test–retest analysis, it was found that the intraclass correlation coefficient (ICC = 0.998, *p* < 0.01) was high, and the scale made consistent measurements over time (Alpar 2020) (Table 4).

**TABLE 4:** Test–retest analysis result of the Professional Fit Scale in Nursing (*n* = 40).

	First application *M* (SD)	Second application *M* (SD)	*t* ^a^	*p* ^a^	*r* ^b^ (ICC)	95% CI		*p* ^b^
						Lower bound	Upper bound	
Professional responsibility	4.26 (0.53)	4.25 (0.52)	1.77	0.08	0.99	0.98	0.99	0.000**
Interpersonal relations and care	4.29 (0.48)	4.28 (0.46)	0.85	0.40	0.98	0.96	0.99	0.000**
Problem solving	4.04 (0.43)	4.01 (0.46)	1.77	0.08	0.97	0.95	0.98	0.000**
Coping with stress	3.59 (0.70)	3.61 (0.69)	−1.52	0.13	0.99	0.98	0.99	0.000**
Professional skills	4.19 (0.68)	4.20 (0.66)	−0.42	0.67	0.97	0.95	0.98	0.000**
Continuous development	3.68 (0.67)	3.61 (0.64)	1.67	0.10	0.92	0.85	0.95	0.000**
Working conditions	3.90 (0.61)	3.89 (0.62)	0.27	0.78	0.97	0.94	0.98	0.000**
Professional achievement	3.50 (0.69)	3.48 (0.66)	1.35	0.18	0.98	0.97	0.99	0.000**
Professional autonomy	4.07 (0.73)	4.06 (0.75)	1.00	0.32	0.99	0.99	0.99	0.000**
Overall scale	4.03 (0.41)	4.03 (0.41)	−1.13	0.26	0.99	0.99	0.99	0.000**

Abbreviations: Avg, average; ICC, intraclass correlation coefficient; SD, standard deviation; *t*, dependent group *t*‐test.

^a^ Dependent groups *t*‐test.

^b^ Pearson's correlation.

** *p* < 0.01.

### 4.4 The Professional Fit Scale in Nursing Scores of the Participants

The overall score average of the participants was found to be 4120 (SD 0.621). The highest average score was obtained from the interpersonal relations and care subscale (4325, SD 0.726), and the lowest score was obtained from the professional achievement subscale (3587, SD 0.958).

### 4.5 Assessment of the Professional Fit Scale in Nursing

The Professional Fit Scale in Nursing, developed within the scope of the current study, and whose validity and reliability analyses have been performed, consists of 56 items and nine subscales. These subscales are as follows: professional responsibility (14 items, 1st–14th), interpersonal relations and care (8 items, 15th–22nd), problem solving (8 items, 23rd–30th), coping with stress (7 items, 31st–37th), professional skills (5 items, 38th–42nd), continuous development (4 items, 43rd–46th), working conditions (4 items, 47th–50th), professional achievement (3 items, 51st–53rd) and professional autonomy (3 items, 54th–56th). The scale does not contain any reversed items.

The scale follows a 5‐point Likert scoring system, where responses range from 1 (strongly disagree) to 5 (strongly agree). The final score is determined by calculating the average of the responses. The average score obtained from the overall scale and subscales that is close to 5 points indicates a high level of professional fit, while those close to 1 point indicate a low level.

## 5 Discussion

The Professional Fit Scale in Nursing was designed as a comprehensive tool that covers various dimensions such as professional responsibility, interpersonal relationships and care, professional skills and coping with stress, and evaluates nurses' fit to the requirements specific to their profession in a multidimensional way. This measure was developed in Turkey, and the findings of this study showed that the Professional Fit Scale in Nursing has strong psychometric properties, showing good factor structure, high internal consistency, and convergent and divergent validity in the expected directions.

The developed scale is specific to the nursing profession. It aims to measure not only nurses' professional responsibilities but also their interpersonal relationships, their ability to cope with stress and their capacity for continuous development. In this respect, it is possible to say that the scale differs from the existing measurement tools in the literature. Associating nurses' professional fit not only with their individual abilities but also with critical areas such as environmental factors (working conditions) and professional autonomy provides an opportunity to evaluate the adaptation process in a broader framework.

Professional fit is an important factor that increases nurses' commitment to their jobs, job satisfaction, and thus the quality of patient care (Meretoja et al. 2004). In this context, it is thought that the scale developed in this study can be a powerful tool to evaluate the extent to which nurses feel themselves suitable for their profession. Especially, the evaluation of sub‐dimensions such as professional success and autonomy may contribute to the understanding of nurses' motivation and professional satisfaction.

This scale can be an important resource for both nursing education and professional development programs. Objectively assessing nurses' professional fit may lead to the structuring of educational curricula and workplace regulations to support this adaptation. As a result, this scale can be considered a tool to be used for improving not only individual nurses but also the entire health ecosystem.

### 5.1 Scale Contents

The Occupational Fit Scale in Nursing, which was developed to evaluate nurses' perceptions of their occupational fit, consists of 9 subscales: Professional responsibility, interpersonal relations and care, problem solving, coping with stress, professional skills, continuous development, working conditions, professional achievement and professional autonomy.

Factor 1 (professional responsibility, 14 items) is a measure of nurses' perceived compliance with various aspects of their professional responsibilities, including job competence, reliability, teamwork, time management, adherence to ethical principles and congruence of personal and professional values. This factor supports studies reporting that relevant professional responsibilities are critical requirements in nursing (Grace 2022; Nabizadeh‐Gharghozar et al. 2021).

Factor 2 (interpersonal relations and care, 8 items) assesses nurses' perceived compliance with important nursing skills such as communication skills, holistic care, and patient‐centered care. This factor is consistent with Watson (1999) Theory of Human Caring, which focuses on holistic, empathic, and compassionate care and emphasizes that care can only be effectively demonstrated and practiced in interpersonal relationships. In addition, this factor reflects previous studies showing that these nursing skills are important skills in improving nursing care, patient satisfaction, treatment adherence and overall health outcomes (Jeong and Seo 2022; Ozan and Okumuş 2017).

Factor 3 (problem solving, 8 items) assesses nurses' perceived compliance with problem‐solving skills such as identifying problems, recognising potential risks, making difficult decisions under risky conditions, and developing different creative solutions to problems. This factor is consistent with other studies that emphasise that problem‐solving skill is a critical requirement for nursing practice and that this skill helps nurses to better serve their patients (Dewi et al. 2021; Mohamad et al. 2022).

Factor 4 (coping with stress, 7 items) describes nurses' perceived compliance with their ability to cope with work‐related stress and factors such as openness to feedback, adaptation to changing conditions and stress management. Preventing and coping with the negative effects of stress is a prerequisite for maintaining high productivity, job satisfaction and a healthy work‐life balance (Antczak‐Komoterska et al. 2023). This factor supports previous studies that emphasise the need for nursing staff to have a variety of coping strategies that allow them to cope with, avoid or minimise work‐related stress (Antczak‐Komoterska et al. 2023; Iwanowicz‐Palus et al. 2022).

Factor 5 (professional skills, 5 items) assesses the nurses' perceived compliance with the ability to use information and communication systems and have sufficient cognitive and psychomotor skills to perform professional practices. These skills are a fundamental part of nursing practice and are critical for improving the quality of patient care, ensuring patient safety, and making effective clinical decisions (Mrayyan et al. 2023; Reid et al. 2022).

Factor 6 (continuous development, 4 items) describes nurses' professional development and continuous learning. This factor reflects previous studies that emphasise the need for nurses to continuously develop and update their competencies to advance in their careers, adapt to changing standards, and provide high‐quality nursing care (Fournier and Fouts 2021; Lee and Lee 2019).

Factor 7 (working conditions, 4 items) assesses nurses' perceived adaptation to the social and physical factors required by nursing jobs that affect nurses' ability to do their jobs, such as working in a shift system, risky work environments and a busy working schedule. This factor coincides with the results of a study by Malinowska‐Lipień et al. (2021) conducted on newly graduated nurses, which emphasised that there is a correlation between work environment characteristics and nurses' views on patient safety, and that this is indirectly related to nurses' willingness to work in challenging environments. In addition, this factor supports existing studies that emphasise the importance of nurses coping with the demands of their profession and working effectively in high‐stress and high‐risk environments (Foster et al. 2023; Ismail et al. 2023).

Factor 8 (professional achievement, 3 items) is related to the ability of nurses to meet their career expectations and to realise their personal goals in the nursing profession and evaluates the compliance perceived by nurses in this direction. This factor supports previous studies emphasising the impact of meeting nurses' career expectations and being able to use their skills in their profession on career satisfaction, professional commitment, turnover intention and nursing practice (Chang et al. 2019; Guerrero et al. 2017).

Factor 9 (professional autonomy, 3 items) assesses nurses' perceived compliance with important aspects of professional autonomy such as their decision‐making ability, independence and level of control. This factor reflects previous studies that emphasise the vital role that professional autonomy in nursing plays in increasing nurses' job satisfaction, maintaining professional continuity and providing safe and quality patient care (Labrague et al. 2019; Ntatseri et al. 2019).

### 5.2 Limitations

Although this study followed the recommended scale development steps, it has some limitations. Due to the COVID‐19 pandemic, online collection of data required nurses to be reached conveniently and by snowball sampling method. Therefore, it limited reaching a larger number of nurses. In addition, the cross‐sectional collection of data at a single time is also a limitation and may have an effect on the findings. Finally, since the Professional Fit Scale in Nursing is a self‐report instrument, the responses reflect subjective perceptions and may be influenced by social desirability or response bias. The findings from the validity and reliability analyses of the scale should be considered the first application results. Further validation studies are recommended in different settings and with larger samples.

## 6 Conclusion

The Professional Fit Scale in Nursing is a measurement tool with adequate psychometric properties that can be used to determine the professional fit of nurses. It can assess the level of professional fit of nurses through self‐reporting and can be adapted to different cultures. The scale, which can be applied to all nurses, can also be used specifically in the recruitment and placement of newly graduated nurses. In accordance with the results to be obtained from the scale, training and development programs aimed at improving the professional fit of nurses at the individual or organisational level can be carried out. The scale can be referred to examine the relationship between nurses' professional fit levels and performance and/or competence assessments, talent management and job satisfaction, and career success/satisfaction. In addition, its relationship with patient outcomes such as patient experience/satisfaction can also be examined.

## Author Contributions

Tuba Çatak and Betül Sönmez made substantial contributions to conception and design, or acquisition of data, or analysis and interpretation of data; involved in drafting the manuscript or revising it critically for important intellectual content; has given final approval of the version to be published. Each author has participated sufficiently in the work to take public responsibility for appropriate portions of the content; and agreed to be accountable for all aspects of the work in ensuring that questions related to the accuracy or integrity of any part of the work are appropriately investigated and resolved.

## Disclosure

Statistical Statement: The authors have checked to make sure that our submission conforms, as applicable, to the Journal's statistical guidelines *described here*. The statistics were checked prior to submission by an expert statistician: Fatih SONTAY spssdestek@gmail.com. The authors affirm that the methods used in the data analyses are suitably applied to their data within their study design and context, and the statistical findings have been implemented and interpreted correctly. The authors agree to take responsibility for ensuring that the choice of statistical approach is appropriate and is conducted and interpreted correctly as a condition to submit to the Journal.

## Ethics Statement

Istanbul University‐Cerrahpaşa Ethics Committee of Social and Humanities Research (Date: 07/09/2020, Decision Number: 53728).

## Conflicts of Interest

The authors declare no conflicts of interest.

## Supporting information


**Appendix S1:** nop270309‐sup‐0001‐AppendixS1.docx.

## Data Availability

The data that support the findings of this study are available from the corresponding author upon reasonable request.
