# Supplementary material for: Development and Validation of the Professional Fit Scale in Nursing
Source: Nurs Open. 2025 Nov 19;12(11):e70309. doi: 10.1002/nop2.70309 (PMC12627925; doi:10.1002/nop2.70309)
Supplement: Supplementary file 1 — Appendix S1: nop270309‐sup‐0001‐AppendixS1.docx. [file NOP2-12-e70309-s001.docx]

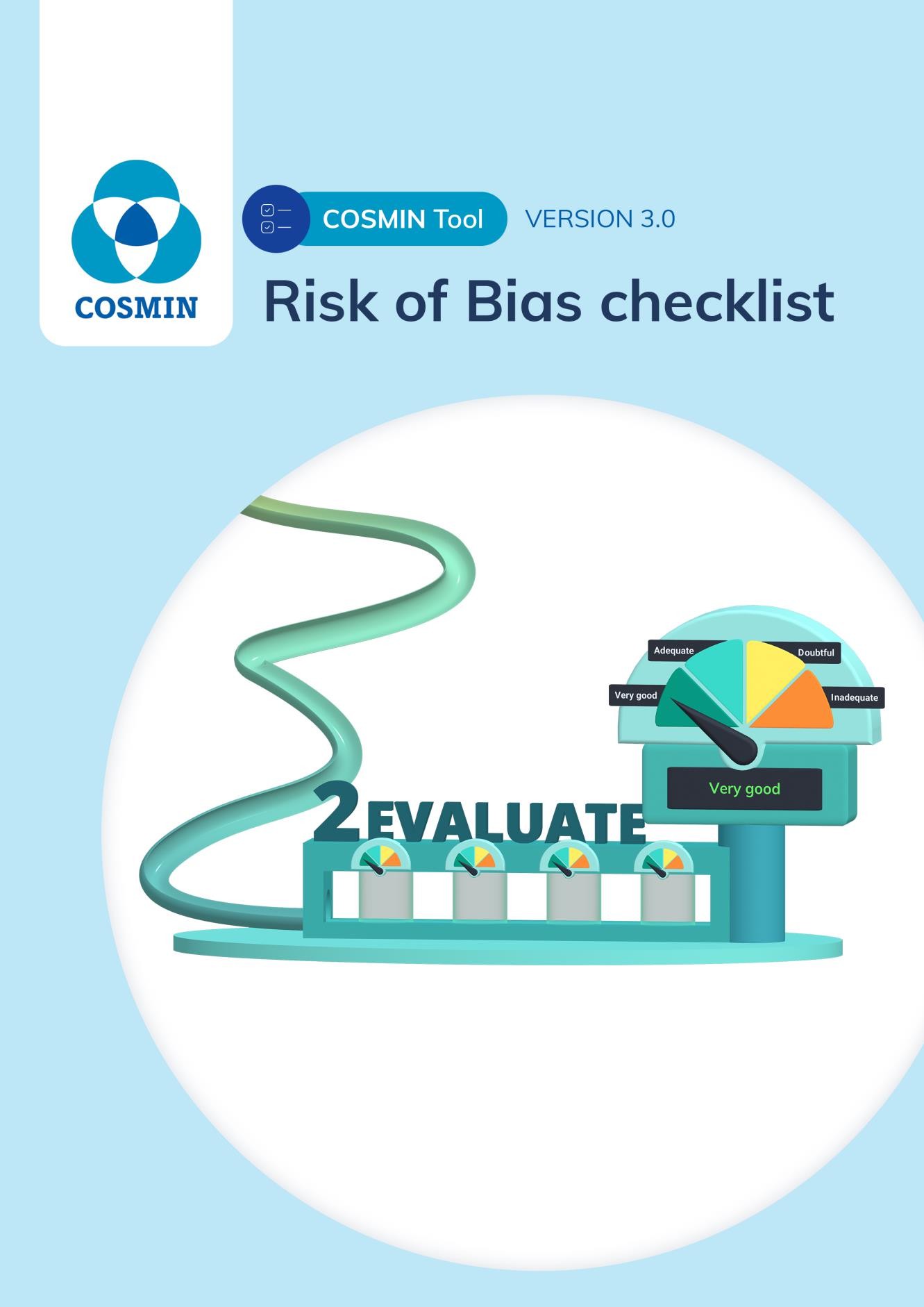


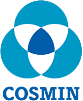


COSMIN Risk of Bias checklist

**Date:** 27 August 2024

**Contact**

L.B. Mokkink, PhD

Amsterdam University Medical Centers Department of Epidemiology and Data Science Amsterdam Public Health research institute

P.O. box 7057

1007 MB Amsterdam The Netherlands Website: [www.cosmin.nl](http://www.cosmin.nl/)

E-mail: [w.mokkink@amsterdamumc.nl](mailto:w.mokkink@amsterdamumc.nl)

*How to site the COSMIN Risk of Bias Checklist*

Please refer to the following study when using the COSMIN Risk of Bias Checklist version 3:

LB Mokkink, E Elsman, CB Terwee. (2024). The COSMIN guideline systematic reviews of Patient-Reported Outcome Measures (PROMs). Qual Life Res. https://doi.org/10.1007/s11136-024-03761-6.

For details on how to use the COSMIN risk of Bias checklist see ‘COSMIN guideline for conducting systematic reviews of Patient-Reported Outcome Measures (PROMs) – user manual’ available from our website [www.cosmin.nl.](http://www.cosmin.nl/)


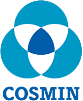


Abbreviations used:

*AUC – area under the receiver operating characteristic curve CTT – classical test theory*

*DIF – differential item functioning FA – factor analysis*

*ICC – intraclass correlation coefficient IRT – item response theory*

*KR-20 - Kuder-Richardson formule 20 LoA – limits of agreement*

*MGCFA – multi-group confirmatory factor analysis MI – measurement invariance*

*NA – not applicable*

*PCA – principal component analysis*

*PROM – patient-reported outcome measure SDC – smallest detectable change*

*SE (θ) – standard error of the theta SEM – standard error of measurement 1PL model – 1 parameter IRT model 2PL model – 2 parameter IRT model*


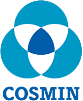


**Instructions**

*Tick the boxes that need to be completed for the article*

|  | **COSMIN Risk of Bias checklist** |
| --- | --- |
|  | Box 1. PROM development |
|  | Box 2. Content validity |
|  | Box 3. Structural validity |
|  | Box 4. Internal consistency |
|  | Box 5. Cross-cultural validity\Measurement invariance |
|  | Box 6. Reliability |
|  | Box 7. Measurement error |
|  | Box 8. Criterion validity |
|  | Box 9. Hypotheses testing for construct validity |
|  | Box 10. Responsiveness |

To assess the methodological quality of each study, i.e. assessing the risk of bias of the result of a study, you can use the corresponding COSMIN Risk of Bias box. You can complete each

standard in the box, and use the ‘worst score counts’ method to determine the overall quality of a study (i.e. by taking the lowest rating of any standard in the box). For example, if for a reliability study one item in a box is rated as ‘inadequate’, the overall methodological quality of that reliability study is rated as ‘inadequate’. The response option ‘NA’ (not applicable) is at issue for some standards. For example, when a study on structural validity is based on CTT, the standard on IRT is not applicable and this standard should not be

considered in the “worst score counts”- rating for that specific study. For standards where this option is not at issue, these cells are grey and shouldn’t be used.


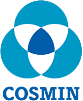


| Scope of the PROM | |  |
| --- | --- | --- |
| 1 | Is a clear description provided of the construct to be measured? | - Construct clearly described - Construct not clearly described |
| 2 | Is the origin of the construct clear: was a theory, conceptual framework or disease model used or clear rationale provided to define the construct to be measured? | - Origin of the construct clear - Origin of the construct not clear |
| 3 | Is a clear description provided of the target population for which the PROM was developed? | - Target population clearly described - Target population not clearly described |
| 4 | Is a clear description provided of the context of use | - Context of use clearly described - Context of use not clearly described |
| 5 | What is the measurement model on which the PROM is based? | - Reflective model - Formative model^1^ - unclear |

^1^ If the scale is not based on a reflective model, unidimensionality or structural validity is not relevant.

| Does the study concern unidimensionality or structural validity? ^2^ | - unidimensionality - structural validity |
| --- | --- |

^2^ In a systematic review, it is helpful to make a distinction between studies where factor analysis is performed on each (sub)scale separately to evaluate whether the (sub)scales are unidimensional (unidimensionality studies) and studies where factor analysis is performed on all items of an instrument to evaluate the (expected) number of subscales in the instrument and the clustering of items within subscales (structural validity studies).

| **Box 1. PROM development** | | | | | | |
| --- | --- | --- | --- | --- | --- | --- |
| *1a. Concept elicitation study (relevance and comprehensiveness)* | | **very good** | **adequate** | **doubtful** | **inadequate** | **NA** |
|  |  |  | | | | |
| 1 | Was the concept elicitation study performed in a sample representing the target population for which the PROM was developed? | Study performed in a sample representing the target population | Assumable that the study was performed in a sample representing the target population, but not clearly described | Doubtful whether the study was performed in a sample representing the target population | Study not performed in a sample representing the target population | NA |
| 2 | Was an appropriate qualitative data collection method used to identify relevant items for a new PROM? | Widely recognized or well justified qualitative method used, suitable for the construct and study population | Assumable that the qualitative method was appropriate and suitable for the construct and study population, but not clearly described | Only quantitative (survey) method(s) used or doubtful whether the method was suitable for the construct and study population | Method used not appropriate or not suitable for the construct or study population |  |
| 3 | Were skilled group moderators/interviewers used? | Skilled group moderators/ interviewers used | Group moderators  /interviewers had limited experience or were trained specifically for the study | Not clear if group moderators  /interviewers were trained or group moderators  /interviewers not trained and no experience |  | NA |

| 4 | Were the group meetings or interviews based on an appropriate topic or interview guide? | Appropriate topic or interview guide | Assumable that the topic or interview guide was appropriate, but not clearly described | Not clear if a topic guide was used or doubtful if topic or interview guide was appropriate or no guide |  | NA |
| --- | --- | --- | --- | --- | --- | --- |
| 5 | Were the group meetings or interviews recorded and transcribed verbatim? | All group meetings or interviews were recorded and transcribed verbatim | Assumable that all group meetings or interviews were recorded and transcribed verbatim, but not clearly described | Not clear if all group meetings of interviews were recorded and transcribed verbatim or recordings not transcribed verbatim or only notes were made during the group meetings/ interviews | No recording and no notes | NA |
| 6 | Was an appropriate approach used to analyse the data? | A widely recognized or well justified approach was used | Assumable that the approach was appropriate, but not clearly described | Not clear what approach was used or doubtful whether the approach was appropriate | Approach not appropriate |  |
| 7 | Was at least part of the data coded independently? | At least 50% of the data was coded by at least two researchers independently | 11-49% of the data was coded by at least two researchers independently | Doubtful if two researchers were involved in the coding or only 1-10% of the data was coded by at least two researchers independently | Only one researcher was involved in coding or no coding | Not applicab le |
| 8 | Was data collection continued until saturation was reached? | Evidence provided that saturation was reached | Assumable that saturation was reached | Doubtful whether saturation was reached | Evidence suggests that saturation was not reached | NA |
| 9 | For quantitative studies (surveys): was the sample size appropriate? | ≥100 | 50-99 | 30-49 | <30 | NA |

| *Other* |  | | | |
| --- | --- | --- | --- | --- |
| 10 Were there any other important flaws in the design or methods of  the study? | No other important methodological flaws |  | Other minor Other important methodological flaws methodological  flaws |  |

| **1b. Pilot study (Cognitive interview study or other pilot test)** (comprehensibility) | | | | | | |
| --- | --- | --- | --- | --- | --- | --- |
|  | | **very good** | **adequate** | **doubtful** | **inadequate** | **NA** |
| *Comprehensibility* | |  | | | | |
| 11 | Was the pilot study performed in a sample representing the target population for which the PROM was developed? | Study performed in a sample representing the target population  AND  Sample was not included in the concept elicitation study | Assumable that the study was performed in a sample representing the target population but not clearly described, or sample was included in the concept elicitation  study | Doubtful whether the study was performed in a sample representing the target population | Study not performed in a sample representing the target population |  |
| 12 | Was the comprehensibility assessed of the PROM instructions, items, response options, and recall period? | Comprehensibility of the PROM instructions, items, response options, and recall period was assessed |  | Not clear if patients were asked about the comprehensibility of all items, response options, instructions, and recall period OR patients were not asked about the comprehensibility of the PROM instructions or the recall period | Patients were not asked about the comprehensibility of all items and response options |  |

1. Were all items tested in their final form?

All items were tested in their final form

Assumable that all items were tested in their final form, but not clearly described

Not clear if all items were tested in their final form

Items were not tested in their final form or items were not re-tested after substantial adjustments

1. Was an appropriate qualitative method used?
2. Was each item tested in an appropriate number of patients?

For qualitative studies

For quantitative (survey) studies

Widely recognized or well justified qualitative method used

≥7

≥50

Assumable that the method was appropriate but not clearly described

4-6

≥30

Only quantitative (survey) method(s) used or doubtful whether the method was appropriate

<4 or not clear

<30 or not clear

Method used not appropriate

1. Were skilled interviewers used?

Skilled interviewers used Interviewers had

limited experience or were trained specifically for the study

Not clear if interviewers NA were trained OR

interviewers were not and had no experience

1. Were the interviews based on an appropriate interview guide?

Appropriate topic or interview guide

Assumable that the topic or interview guide was appropriate, but not clearly described

Not clear if a topic guide NA was used or doubtful if

topic or interview guide was appropriate OR no guide

1. Were the interviews recorded and transcribed verbatim?

All interviews were recorded and transcribed verbatim

Assumable that all interviews were recorded and transcribed verbatim, but not clearly described

Not clear if all interviews were recorded and transcribed verbatim OR recordings not transcribed verbatim OR only notes were made during the interviews

No recording and NA no notes

| 19 | Was an appropriate approach used to analyse the data? | A widely recognized or well justified approach was used | Assumable that the approach was appropriate, but not clearly described | Not clear what approach was used OR doubtful whether the approach was appropriate | Approach not appropriate |  |
| --- | --- | --- | --- | --- | --- | --- |
| 20 | Were at least two researchers involved in the analysis? | At least two researchers involved in the analysis | Assumable that at least two researchers were involved in the analysis, but not clearly described | Not clear if two researchers were included in the analysis OR only one researcher involved in the analysis |  |  |
| 21 | Were problems regarding the comprehensibility of the PROM instructions, items, response options, and recall period appropriately addressed by adapting the PROM? | No problems found OR problems appropriately addressed and PROM was adapted and re-tested if necessary | Assumable that there were no problems or that problems were appropriately addressed, but not clearly described | Not clear if there were problems OR doubtful if problems were appropriately addressed | Problems not appropriately addressed OR PROM was adapted but items were not re-tested after substantial adjustments | NA |
| *Other* | |  |  |  |  |  |
| 22 | Were there any other important flaws in the design or  methods of the study? | No other important methodological flaws |  | Other minor methodological flaws | Other important methodological  flaws |  |

| **Box 2. Content validity**  **2a. Asking patients about relevance** | | | | | | |
| --- | --- | --- | --- | --- | --- | --- |
| *Design requirements* | | **very good** | **adequate** | **doubtful** | **inadequate** | **NA** |
| 1 | Was an appropriate method used to ask patients whether each item is relevant for their experience with the condition?  Was each item tested in an appropriate number of patients?  For qualitative studies  For quantitative (survey) studies  Were skilled group moderators/interviewers used?  Were the group meetings or interviews based on an appropriate topic or interview guide? | Widely recognized or well justified method used | Only quantitative (survey) method(s) used or assumable that the method was appropriate but not clearly  described | Not clear if patients were asked whether each item is relevant OR doubtful whether the method was appropriate | Method used not appropriate OR patients not asked about the relevance of all items |  |
| 2 |  |  |  |  |  |  |
|  |  | ≥7  ≥50 | 4-6  ≥30 | <4 or not clear  <30 or not clear |  |  |
| 3 |  | Skilled group moderators/ interviewers used | Group moderators  /interviewers had limited experience or were trained specifically for the study | Not clear if group moderators /interviewers were trained OR group moderators /interviewers were not trained and had no experience |  | NA |
| 4 |  | Appropriate topic or interview guide | Assumable that the topic or interview guide was appropriate, but not clearly described | Not clear if a topic guide was used OR doubtful if topic or interview guide was appropriate OR no guide |  | NA |

| 5 | Were the group meetings or interviews recorded and transcribed verbatim? | All group meetings or interviews were recorded and transcribed verbatim | Assumable that all group meetings or interviews were recorded and transcribed verbatim, but not clearly described | Not clear if all group meetings or interviews were recorded and transcribed verbatim OR recordings not transcribed verbatim OR only notes were made during the group meetings/ interviews | No recording and no notes | NA |
| --- | --- | --- | --- | --- | --- | --- |
| *Analyses* | |  |  |  |  |  |
| 6 | Was an appropriate approach used to analyse the data? | A widely recognized or well justified approach was used | Assumable that the approach was appropriate, but not clearly described | Not clear what approach was used OR doubtful whether the approach was appropriate | Approach not appropriate |  |
| 7 | Were at least two researchers involved in the analysis? | At least two researchers involved in the analysis | Assumable that at least two researchers were involved in the analysis, but not  clearly described | Not clear if two researchers were included in the analysis OR only one researcher involved in the analysis |  |  |
| *Other* | |  |  |  |  |  |
| 8 | Were there any other important flaws in the design or methods of the study? | No other important methodological  flaws |  | Other minor methodological flaws | Other important methodological flaws |  |

| **2b Asking patients about comprehensiveness** | | | | | | |
| --- | --- | --- | --- | --- | --- | --- |
| *Design requirements* | | **very good** | **adequate** | **doubtful** | **inadequate** | **NA** |
| 9 | Was an appropriate method used for assessing the comprehensiveness of the PROM?  Was the PROM tested in an appropriate number of patients?  For qualitative studies  For quantitative (survey) studies  Were skilled group moderators/interviewers used?  Were the group meetings or interviews based on an appropriate topic or interview guide? | Widely recognized or well justified method used | Only quantitative (survey) method(s) used OR assumable that the method was appropriate but not clearly described | Doubtful whether the method was appropriate | Method used not appropriate |  |
| 10 |  |  |  |  |  |  |
|  |  | ≥7  ≥50 | 4-6  ≥30 | <4 or not clear  <30 or not clear |  |  |
| 11 |  | Skilled group moderators/ interviewers used | Group moderators  /interviewers had limited experience or were trained specifically for the study | Not clear if group moderators /interviewers were trained OR group moderators /interviewers were not trained and had no experience |  | NA |
| 12 |  | Appropriate topic or interview guide | Assumable that the topic or interview guide was appropriate, but not clearly described | Not clear if a topic guide was used OR doubtful if topic or interview guide was appropriate OR no guide |  | NA |

| 13 | Were the group meetings or interviews recorded and transcribed verbatim? | All group meetings or interviews were recorded and transcribed verbatim | Assumable that all group meetings or interviews were recorded and transcribed verbatim, but not clearly described | Not clear if all group meetings or interviews were recorded and transcribed verbatim OR recordings not transcribed verbatim OR only notes were made during the  group meetings/ interviews | No recording and no notes | NA |
| --- | --- | --- | --- | --- | --- | --- |
| *Analyses* | |  |  |  |  |  |
| 14 | Was an appropriate approach used to analyse the data? | A widely recognized or well justified approach was used | Assumable that the approach was appropriate, but not clearly described | Not clear what approach was used OR doubtful whether the approach was appropriate | Approach not appropriate |  |
| 15 | Were at least two researchers involved in the analysis? | At least two researchers involved in the analysis | Assumable that at least two researchers were involved in the analysis, but not  clearly described | Not clear if two researchers were included in the analysis OR only one researcher involved in the analysis |  |  |
| *Other* | |  |  |  |  |  |
| 16 | Were there any other important flaws in the design or methods of the study? | No other important methodological flaws |  | Other minor methodological flaws | Other important methodological flaws |  |

| **2c Asking patients about comprehensibility** | | | | | | |
| --- | --- | --- | --- | --- | --- | --- |
| *Design requirements* | | **very good** | **adequate** | **doubtful** | **inadequate** | **NA** |
| 17 | Was an appropriate qualitative method used for assessing the comprehensibility of the PROM instructions, items, response options, and recall period? | Widely recognized or well justified qualitative method used | Assumable that the method was appropriate but not clearly described | Only quantitative (survey) method(s) used OR doubtful whether the method was appropriate OR not clear if patients were asked about the comprehensibility of the items, response options and recall period OR patients not asked about the comprehensibility of the PROM instructions or  recall period | Method used not appropriate OR patients not asked about the comprehensibilit y of the items, response options, and recall period |  |
| 18 | Was each item tested in an appropriate number of patients?  For qualitative studies  For quantitative (survey) studies | ≥7  ≥50 | 4-6  ≥30 | <4 or not clear  <30 or not clear |  |  |
| 19 | Were skilled group moderators/interviewers used? | Skilled group moderators/ interviewers used | Group moderators  /interviewers had limited experience or were trained specifically for the study | Not clear if group moderators /interviewers were trained OR group moderators /interviewers were not trained and had no experience |  |  |
| 20 | Were the group meetings or interviews based on an appropriate topic or interview guide? | Appropriate topic or interview guide | Assumable that the topic or interview guide was appropriate, but not clearly described | Not clear if a topic guide was used or doubtful if topic OR interview guide was appropriate OR no guide |  | NA |

| 21 | Were the group meetings or interviews recorded and transcribed verbatim? | All group meetings or interviews were recorded and transcribed verbatim | Assumable that all group meetings or interviews were recorded and transcribed verbatim, but not clearly described | Not clear if all group meetings or interviews were recorded and transcribed verbatim OR recordings not transcribed verbatim OR only notes were made during the  group meetings/ interviews | No recording and no notes | NA |
| --- | --- | --- | --- | --- | --- | --- |
| *Analyses* | |  |  |  |  |  |
| 22 | Was an appropriate approach used to analyse the data? | A widely recognized or well justified approach was used | Assumable that the approach was appropriate, but not clearly described | Not clear what approach was used OR doubtful whether the approach was appropriate | Approach not appropriate |  |
| 23 | Were at least two researchers involved in the analysis? | At least two researchers involved in the analysis | Assumable that at least two researchers were involved in the analysis, but not  clearly described | Not clear if two researchers were included in the analysis OR only one researcher involved in the analysis |  |  |
| *Other* | |  |  |  |  |  |
| 24 | Were there any other important flaws in the design or methods of the study? | No other important methodological flaws |  | Other minor methodological flaws | Other important methodologic al flaws |  |

| **2d. Asking professionals about relevance** | | | | |
| --- | --- | --- | --- | --- |
| *Design requirements* | **very good** | **adequate** | **doubtful inadequate** | **NA** |
| 1. Was an appropriate method used to ask professionals whether each item is relevant for the construct of interest? 2. Were professionals from all relevant disciplines included? 3. Was each item tested in an appropriate number of professionals? For qualitative studies   For quantitative (survey) studies  *Analyses*   1. Was an appropriate approach used to analyse the data? | Widely recognized or well justified method used | Only quantitative (survey) method(s) used OR assumable that the method was appropriate but not clearly described | Not clear if Method used professionals were not  asked whether appropriate OR each item is professionals  relevant OR not asked doubtful whether about the  the method was relevance of all appropriate items | |
|  | Professionals from all required disciplines were included | Assumable that professionals from all required disciplines were included, but not clearly described | Doubtful whether professionals from all required disciplines were included OR relevant professionals were not included | |
|  | ≥7  ≥50 | 4-6  ≥30 | <4 or not clear  <30 or not clear | |
|  | A widely recognized or well justified approach was used | Assumable that the approach was appropriate, but not clearly described | Not clear what Approach not approach was used appropriate OR doubtful  whether the approach was appropriate | |

| 29 | Were at least two researchers involved in the analysis? | At least two researchers involved in the analysis | Assumable that at least two researchers were involved in the analysis, but not clearly described | Not clear if two researchers were included in the analysis OR only one researcher involved in the analysis |  |
| --- | --- | --- | --- | --- | --- |
| *Other* | |  |  |  |  |
| 30 | Were there any other important flaws in the design or methods of the study? | No other important methodological flaws |  | Other minor methodological flaws | Other important methodological flaws |

| **2e. Asking professionals about comprehensiveness** | | | | | | |
| --- | --- | --- | --- | --- | --- | --- |
| *Design requirement* | | **very good** | **adequate** | **doubtful** | **inadequate** | **NA** |
|  | Was an appropriate method used for assessing the comprehensiveness of the PROM? |  | | | | |
| 31 |  | Widely recognized or well justified method used | Only quantitative (survey) method(s) used OR assumable that the method was appropriate but not clearly described | Doubtful whether the method was appropriate | Method used not appropriate |  |
| 32 | Were professionals from all relevant disciplines included? | Professionals from all required disciplines were included | Assumable that professionals from all required disciplines were included, but not clearly described | Doubtful whether professionals from all required disciplines were included OR relevant professionals were not included |  |  |

| 1. Was the PROM tested in an appropriate number of professionals? For qualitative studies   For quantitative (survey) studies  *Analyses*   1. Was an appropriate approach used to analyse the data? 2. Were at least two researchers involved in the analysis?   *Other*   1. Were there any other important flaws in the design or methods of the study? | ≥7  ≥50 | 4-6  ≥30 | <4 or not clear  <30 or not clear |
| --- | --- | --- | --- |
|  | A widely recognized or well justified approach was used | Assumable that the approach was appropriate, but not clearly described | Not clear what Approach not approach was used appropriate OR doubtful  whether the approach was appropriate |
|  | At least two researchers involved in the analysis | Assumable that at least two researchers were involved in the analysis, but not clearly described | Not clear if two researchers were included in the analysis OR only one researcher involved in the analysis |
|  | No other important methodological flaws |  | Other minor Other important methodological methodological flaws flaws |

| **2f. Asking professionals about comprehensibility** | | | | | | |
| --- | --- | --- | --- | --- | --- | --- |
| *Design requirement* | | **very good** | **adequate** | **doubtful** | **inadequate** | **NA** |
|  | Was an appropriate method used for assessing the comprehensibility of the PROM instructions, items, response options, and recall period? |  | | | | |
| 37 |  | Widely recognized or well justified method used | Assumable that the method was appropriate but not clearly described | Only quantitative (survey) method(s) used OR doubtful whether the method was appropriate OR not clear if professionals were asked about the comprehensibility of all items, response options instructions, and recall period OR professionals were not asked about the comprehensibility of the PROM instructions or the recall period | Method used not appropriate OR professionals not asked about the comprehensibil ity of all items and response options |  |
| 38 | Were professionals from all relevant disciplines included? | Professionals from all required disciplines were included | Assumable that professionals from all required disciplines were included, but not clearly described | Doubtful whether professionals from all required disciplines were included OR relevant professionals were not included |  |  |
| 39 | Was each item tested in an appropriate number of professionals?  For qualitative studies  For quantitative (survey) studies | ≥7  ≥50 | 4-6  ≥30 | <4 or not clear  <30 or not clear |  |  |

| *Analyses* |  |  |  |
| --- | --- | --- | --- |
| 40 Was an appropriate approach used to analyse the data? | A widely recognized or well justified approach was used | Assumable that the approach was appropriate, but not clearly described | Not clear what approach was Approach not used OR doubtful whether appropriate the approach was  appropriate |
| 41 Were at least two researchers involved in the analysis? | At least two researchers involved in the analysis | Assumable that at least two researchers were involved in the analysis, but not  clearly described | Not clear if two researchers were included in the analysis OR only one researcher involved in the analysis |
| *Other* |  |  |  |
| 42 Were there any other important flaws in the design or methods of the study? | No other important methodological flaws |  | Other minor methodological Other important flaws methodological  flaws |

| **Box 3. Structural validity** | | | | |
| --- | --- | --- | --- | --- |
| *Statistical methods* | **very good** | **adequate doubtful** | **inadequate** | **NA** |
| 1 For CTT: Was exploratory or confirmatory factor analysis | Confirmatory factor analysis performed  Chosen model fits well to the research question  FA: 7 times the number of items in the tested model and ≥100  Rasch/1PL models: ≥ 200  subjects  2PL parametric IRT models OR Mokken scale analysis: ≥ 1000 subjects  No other important methodological flaws | Exploratory factor Only PCA was  analysis performed performed  Assumable that the Doubtful if the chosen model fits well to chosen model fits the research question well to the  research question  FA: at least 5 times the FA: 5 times the number of items in the number of items in tested model and ≥100; the tested model OR at least 6 times but <100  number of items in the tested model but <100  Rasch/1PL models: 100- Rasch/1PL models: 199 subjects 50-99 subjects  2PL parametric IRT 2PL parametric IRT models OR Mokken scale models OR Mokken analysis: 500-999 scale analysis: 250-  subjects 499 subjects  Other minor methodological flaws (e.g. rotation method not described) | No exploratory or confirmatory factor analysis performed  Chosen model does not fit to the research question  FA: < 5 times the number of items in the tested model  Rasch/1PL models: < 50 subjects  2PL parametric IRT models OR Mokken scale analysis: < 250 subjects  Other important methodological flaws (e.g. inappropriate rotation method) | NA  NA |
| performed? |  |  |  |  |
| 2 For IRT/Rasch: does the chosen model fit to the research |  |  |  |  |
| question? |  |  |  |  |
| 3 Was the sample size included in the analysis adequate? |  |  |  |  |
| *Other* |  |  |  |  |
| 4 Were there any other important flaws in the design or |  |  |  |  |
| statistical methods of the study? |  |  |  |  |

| **Box 4. Internal consistency** | | | | | |
| --- | --- | --- | --- | --- | --- |
| *Statistical methods* | **very good** | **adequate** | **doubtful** | **inadequate** | **NA** |
| 1 For continuous scores: Was Cronbach’s alpha or omega  calculated? | Cronbach’s alpha, or  Omega calculated | Only item-total correlations calculated | | No Cronbach’s alpha and no item-total correlations calculated | NA |
| 2 For dichotomous scores: Was Cronbach’s alpha or KR-20 calculated? | Cronbach’s alpha or KR- 20 calculated | Only item-total correlations calculated | | No Cronbach’s alpha or KR-20 and no item-total correlations calculated | NA |
| 3 For IRT-based scores: Was standard error of the theta (SE (θ)) or reliability coefficient of estimated latent trait value (index of (subject or item) separation) calculated? | SE(θ) or reliability coefficient calculated |  | | SE(θ) or reliability coefficient NOT calculated | NA |
| *Other* |  |  | |  |  |
| 4 Were there any other important flaws in the design or statistical methods of the study? | No other important methodological flaws | Other minor methodological flaws | | Other important methodological flaws |  |

| **Box 5. Cross-cultural validity\Measurement invariance** | | | | | |
| --- | --- | --- | --- | --- | --- |
| *Design requirements* | **very good** | **adequate** | **doubtful** | **inadequate** | **NA** |
| 1 Were the samples similar for relevant characteristics except for the group variable? | Evidence provided that samples were similar for relevant characteristics except group variable | Stated (but no evidence provided) that samples were similar for relevant characteristics except  group variable | Unclear whether samples were similar for relevant characteristics except group variable | Samples were NOT similar for relevant characteristics except group variable |  |
| *Statistical methods* |  |  |  |  |  |
| 2 Was an appropriate approach used to analyse the data? | A widely recognized or well justified approach was used | Assumable that the approach was appropriate, but not clearly described | Not clear what approach was used OR doubtful whether the approach was appropriate | Approach not appropriate | NA |
| 3 Was the sample size included in the analysis adequate? | MGCFA: 7 times the number of items in the model and ≥100  Regression analyses or IRT/Rasch based analyses: 200 subjects per group | 5 times the number of items in the model and ≥100; OR 5-7 times the number of items in the model but <100  150 subjects per group | 5 times the number of items in the model but <100  100 subjects per group | <5 times the number of items in the model  < 100 subjects per group |  |
| *Other* |  |  |  |  |  |
| 4 Were there any other important flaws in the design or statistical methods of the study? | No other important methodological flaws |  | Other minor methodological flaws | Other important methodological flaws |  |

| **Box 6. Reliability** | | | | | |
| --- | --- | --- | --- | --- | --- |
| *Design requirements* | **very good** | **adequate** | **doubtful** | **inadequate** | **NA** |
| 1 Were patients stable on the construct to be measured in the time between the repeated measurements? | Evidence provided that patients were stable | Assumable that patients were stable | Unclear if patients were stable | Patients were NOT stable |  |
| 2 Was the time interval between the repeated measurements appropriate? | Time interval appropriate |  | Doubtful if time interval was appropriate OR time interval was not stated | Time interval NOT appropriate |  |
| 3 Were the measurement conditions similar for the repeated measurements – except for the condition being evaluated? | Measurement conditions were similar (evidence provided) | Assumable that measurement conditions were  similar | Unclear if measurement conditions were  similar | Measurement conditions were NOT similar |  |
| *Statistical methods* |  |  |  |  |  |
| 4 For continuous scores: Was the appropriate intraclass correlation coefficient (ICC) calculated? | ICCagreement was or can be calculated | (ICCconsistency, Pearson or Spearman correlation coefficient was calculated, OR the ICC model or formula was not described)  WITH  evidence provided that no systematic change between measurements has occurred  OR ICCone-way was calculated | (ICCconsistency, Pearson or Spearman correlation coefficient was calculated  OR the ICC model or formula was not described)  WITHOUT evidence provided that no systematic change between measurements has occurred | (ICCconsistency, Pearson or Spearman correlation coefficient was calculated OR the ICC model or formula was not described)  WITH evidence provided that a systematic change between measurements has occurred | NA |

| 5 | For dichotomous scores: was kappa calculated? | Kappa calculated |  |  | NA |
| --- | --- | --- | --- | --- | --- |
| 6 | For nominal scores: was an unweighted kappa calculated? | Unweighted kappa calculated |  |  | NA |
| 7 | For ordinal scores: was a weighted kappa calculated? | Weighted kappa calculated and the weighting scheme was  described | Kappa calculated, but weighting scheme not described | Unweighted Kappa calculated or unclear if weighting was  applied | NA |
| *Other* | |  |  |  |  |
| 8 | Were there any other important flaws in the design or statistical methods of the study? | No other important methodological flaws |  | Other minor methodological flaws | Other important methodological flaws |

| **Box 7. Measurement error** | | | | | |
| --- | --- | --- | --- | --- | --- |
| *Design requirements* | **very good** | **adequate** | **doubtful** | **Inadequate** | **NA** |
| 1 Were patients stable on the construct to be measured in the time between the repeated measurements? | Evidence provided that patients were stable | Assumable that patients were stable | Unclear if patients were stable | Patients were NOT stable | |
| 2 Was the time interval between the repeated measurements appropriate? | Time interval appropriate |  | Doubtful if time interval was appropriate OR time interval was not stated | Time interval NOT appropriate | |
| 3 Were the measurement conditions similar for the measurements –  except for the condition being evaluated as a source of variation? | Measurement conditions were similar (evidence provided) | Assumable that measurement conditions were similar | Unclear if measurement conditions were similar | Measurement conditions were NOT similar | |

| *Statistical methods* | | | | | | |
| --- | --- | --- | --- | --- | --- | --- |
| 4 | For continuous scores: was the Standard Error of Measurement (SEM), Smallest Detectable Change (SDC) or Limits of Agreement (LoA) calculated? | SEMagreement, SEMone- way, SDCagreement,  or SDCone-way was or could be calculated | (SEMconsistency or SDCconsistency or LoA was calculated  OR the SEM/SDC model or formula was not described)  WITH evidence provided that no systematic change between the measurement has occurred | (SEMconsistency, SDCconsistency, or LoA was calculated)  WITHOUT evidence provided that no systematic change between measurements has occurred | SEM calculated based on Cronbach’s alpha or  SD from another population  OR  (SEMconsistency SDCconsistency or LoA calculated )  WITH evidence provided that systematic change has occurred | NA |
| 5 | For dichotomous/nominal/ordinal scores: was the percentage (positive and negative) agreement calculated? | % positive and negative agreement  calculated | % agreement calculated |  |  | NA |
| *Other* | |  |  |  |  |  |
| 6 | Were there any other important flaws in the design or statistical methods of the study? | No other important methodological flaws |  | Other minor methodological flaws | Other important methodological flaws |  |

| **Box 8. Criterion validity** | | | | | |
| --- | --- | --- | --- | --- | --- |
|  | **very good** | **adequate** | **doubtful** | **inadequate** | **NA** |
| *Statistical methods* |  |  |  |  |  |
| 1 For continuous scores: were correlations, or the AUC calculated? | Correlations or AUC calculated |  |  |  | NA |
| 2 For dichotomous scores: were sensitivity and specificity determined? | Sensitivity and specificity calculated |  |  |  | NA |
| *Other* |  |  |  |  |  |
| 3 Were there any other important flaws in the design or statistical methods of the study? | No other important methodological flaws |  | Other minor methodological flaws | Other important methodological flaws |  |

| **Box 9. Hypotheses testing for construct validity** | | | | | | |
| --- | --- | --- | --- | --- | --- | --- |
| **9a. Comparison with other outcome measurement instruments (convergent validity)** | | | | | | |
| *Design requirements* | | **very good** | **adequate** | **doubtful** | **inadequate** | **NA** |
|  |  |  | | | | |
| 1 | Is it clear what the comparator instrument(s) measure(s)? | Constructs measured by the comparator instrument(s) is clear |  | | Constructs measured by the comparator instrument(s) is not clear |  |
| 2 | Were the measurement properties of the comparator instrument(s) sufficient? | Sufficient measurement properties of the comparator instrument(s) in a population similar to the study population | Sufficient measurement properties of the comparator instrument(s) but not sure if these apply to the study population | Some information on measurement properties of the comparator instrument(s) in any study population | No information on the measurement properties of the comparator instrument(s), OR evidence for insufficient measurement properties of the comparator  instrument(s) |  |
| *Statistical methods* | |  | | | | |
| 3 | Were statistical methods adequate for the comparisons made? | Statistical methods applied were appropriate | Assumable that statistical methods were appropriate | Statistical methods applied NOT optimal | Statistical methods applied NOT appropriate |  |
| *Other* | |  | | | | |
| 4 | Were there any other important flaws in the design or statistical methods of the study? | No other important methodological flaws |  | Other minor methodological flaws | Other important methodological flaws |  |

| **9b. Comparison between subgroups (discriminative or known-groups validity)** | | | | | | |
| --- | --- | --- | --- | --- | --- | --- |
| *Design requirements* | | **very good** | **adequate** | **doubtful** | **inadequate** | **NA** |
|  | |  |  |  |  |  |
| 5 | Was an adequate description provided of important characteristics of the subgroups? | Adequate description of the important characteristics of the subgroups | Adequate description of most of the important characteristics of the subgroups | Poor of no description of the important characteristics of the subgroups |  |  |
| *Statistical methods* | |  |  |  |  |  |
| 6 | Were statistical methods appropriate for the subgroups being compared? | Statistical methods  applied were appropriate | Assumable that  statistical methods were appropriate | Statistical methods applied NOT optimal | Statistical methods  applied NOT appropriate |  |
| *Other* | |  |  |  |  |  |
| 7 | Were there any other important flaws in the design or statistical methods of the study? | No other important methodological flaws |  | Other minor methodological flaws | Other important methodological flaws |  |

| **Box 10. Responsiveness** | | | | | | |
| --- | --- | --- | --- | --- | --- | --- |
| **10a. Criterion approach (i.e. comparison to a gold standard)** | | | | | | |
|  | | **very good** | **adequate** | **doubtful** | **inadequate** | **NA** |
| *Statistical methods* | |  |  |  |  |  |
| 1 | For continuous scores: were correlations between change scores, or the AUC calculated? | Correlations or AUC calculated |  |  |  | NA |
| 2 | For dichotomous scales: were sensitivity and specificity (changed versus not changed) determined? | Sensitivity and specificity calculated |  |  |  | NA |
| *Other* | |  |  |  |  |  |
| 3 | Were there any other important flaws in the design or statistical methods of the study? | No other important methodological flaws |  | Other minor methodological flaws | Other important methodological flaws |  |

| **10b. Construct approach (i.e. hypotheses testing; comparison with other outcome measurement instruments)** | | | | | | |
| --- | --- | --- | --- | --- | --- | --- |
| *Design requirements* | | **very good** | **adequate** | **doubtful** | **inadequate** | **NA** |
|  |  |  | | | | |
| 4 | Is it clear what the comparator instrument(s) measure(s)? | Constructs measured by the comparator instrument(s) is clear |  | | Constructs measured by the comparator instrument(s) is not clear |  |
| 5 | Were the measurement properties of the comparator instrument(s) sufficient? | Sufficient measurement properties of the comparator instrument(s) in a population similar to the study population | Sufficient measurement properties of the comparator instrument(s) but not sure if these apply to the study population | Some information on measurement properties of the comparator instrument(s) in any study population | NO information on the measurement properties of the comparator instrument(s) OR evidence of poor quality of comparator  instrument(s) |  |
| *Statistical methods* | |  | | | | |
| 6 | Were statistical methods appropriate for the comparisons being made? | Statistical methods applied appropriate | Assumable that statistical methods  were appropriate | Statistical methods applied NOT optimal | Statistical methods applied NOT  appropriate |  |
| *Other* | |  | | | | |
| 7 | Were there any other important flaws in the design or statistical methods of the study? | No other important methodological flaws |  | Other minor methodological flaws | Other important methodological flaws |  |

| **10c. Construct approach: (i.e. hypotheses testing: comparison between subgroups)** | | | | | | |
| --- | --- | --- | --- | --- | --- | --- |
| *Design requirements* | | **very good** | **adequate** | **doubtful** | **inadequate** | **NA** |
|  | |  |  |  |  |  |
| 8 | Was an adequate description provided of important characteristics of the subgroups? | Adequate description of the important characteristics of the  subgroups | Adequate description of most of the important characteristics of the  subgroups | Poor or no description of the important characteristics of the subgroups |  |  |
| *Statistical methods* | |  |  |  |  |  |
| 9 | Were statistical methods appropriate for the subgroups being compared? | Statistical methods applied appropriate | Assumable that statistical methods  were appropriate | Statistical method applied NOT optimal | Statistical method applied NOT  appropriate |  |
| *Other* | |  |  |  |  |  |
| 10 | Were there any other important flaws in the design or statistical methods of the study? | No other important methodological  flaws |  | Other minor methodological flaws | Other important methodological  flaws |  |

| **10d. Construct approach: (i.e. hypotheses testing: before and after intervention)** | | | | | | |
| --- | --- | --- | --- | --- | --- | --- |
| *Design requirements* | | **very good** | **adequate** | **doubtful** | **inadequate** | **NA** |
|  |  |  | | | | |
| 11 | Was an adequate description provided of the intervention given? | Adequate description of the intervention |  | Poor description of the intervention | NO description of the intervention |  |
| *Statistical methods* | |  | | | | |
| 12 | Were statistical methods appropriate for the before-after comparison being made? | Statistical methods applied appropriate | Assumable that statistical methods  were appropriate | Statistical methods applied NOT optimal | Statistical methods applied NOT  appropriate |  |
| *Other* | |  | | | | |
| 13 | Were there any other important flaws in the design or statistical methods of the study? | No other important methodological flaws |  | Other minor methodological flaws | Other important methodological flaws |  |
